# Neurorights: comparing some perspectives on legal reform in global south to analyze the Brazilian case

**DOI:** 10.3389/fnhum.2026.1873948

**Published:** 2026-07-06

**Authors:** Alex Moreira, Murilo Vilaça

**Affiliations:** 1 Fundação Oswaldo Cruz (Fiocruz), Rio de Janeiro, Brazil; 2 Escola Nacional de Saúde Pública, Fundação Oswaldo Cruz, Rio de Janeiro, Brazil

**Keywords:** Brazilian context, neural data, neurorights, neurotechnology, regulation

## Abstract

This paper examines the emergence of neurorights as a legal response to the normative risks associated with the development of neurotechnologies. After defining the concept and its core elements, we adopt a selective comparative perspectives on global south regulatory pathways. It analyzes the Chilean constitutional reform as a pioneering but contested experience, contrasts it with the Brazilian scenario of still-fragmented legislative initiatives, and incorporates the Mexican debate on a general law on neurorights and neurotechnologies and the Argentine discussion on conceptual, procedural, and forensic limits. The article argues that the current Brazilian legal framework provides relevant but insufficient protection against risks such as mind reading, unauthorized neural monitoring, mental-state inference, cognitive manipulation, and the procedural misuse of neural evidence. It further argues that the Brazilian debate should not simply reproduce the Chilean formula, but should instead combine reinterpretations of existing constitutional guarantees with targeted statutory innovation, informed by inter-American principles, PARLATINO’s model law, and UNESCO guidance. The conclusion is that a future Brazilian framework must be comparatively informed, conceptually restrained, and institutionally adapted to the country’s constitutional and regulatory architecture.

## 1 Introduction

The rapid development of neurotechnologies challenges traditional legal categories because these tools no longer merely mediate the relationship between the individual and the external world; rather, they act directly upon the neural substrates of thought, emotion, memory, and volition. Instead of operating solely on observable behaviors or ordinary information flows, new brain–computer interfaces, neuromodulation techniques, and algorithmic systems integrated with the brain now intervene in the material basis of subjective experience, generating unprecedented risks to mental autonomy and to the very legal structure of personhood (Chenier et al., 2023).

The warnings in the literature are consistent: the convergence of neurotechnology and artificial intelligence creates a scenario in which it becomes possible to infer mental states, profile cognitive preferences, and, in certain contexts, modulate neural mechanisms associated with intentions, emotions, and decisions. Although these technologies offer significant therapeutic benefits, particularly in rehabilitation, assisted communication, and the treatment of neurological disorders, they also render the boundary between the inner sphere and the techno-economic environment more permeable, thereby calling into question the inviolability of psychic life (Yuste et al., 2017).

In light of this context, neurorights emerge as a normative response to the need to protect the mental sphere against unprecedented forms of extraction, processing, and intervention. The debate is not limited to extending already established legal frameworks; rather, it requires rethinking the adequacy of categories such as privacy, consent, physical integrity, and data protection when the protected object becomes brain activity and the information derived from it (Ienca, 2021).

However, the emergence of neurorights remains controversial. A growing body of literature warns that neurotechnological risks do not automatically justify the proliferation of entirely new human rights, since part of the protected interests may already be covered by privacy, freedom of thought, mental integrity, and personality rights. From this perspective, the central legal question is not only whether the mind deserves heightened protection, but also whether such protection should be achieved through new rights, through the reconceptualization of existing rights, or through a hybrid regulatory strategy combining both pathways (López-Silva and Madrid, 2021; Zúñiga Fajuri et al., 2020; Borbón et al., 2020; Bublitz, 2022; Hertz, 2023; Muñoz and Marinaro, 2023; Brown, 2024).

This article is structured in five parts and adopts a selective comparative-legal strategy rather than an exhaustive survey of all jurisdictions discussing neurorights. First, it conceptually defines neurorights and their core elements. Second, it examines the Chilean experience, while also addressing the main criticisms directed at its constitutional reform. Third, it broadens the comparison by considering other Latin American pathways, especially the Mexican proposals and the Argentine experience at both federal and provincial levels. Fourth, it presents the Brazilian scenario, still marked by ongoing legislative initiatives and by the coexistence of constitutional, civil, data-protection, and internet-law techniques. Finally, it argues that the Brazilian legal framework, although relevant as a starting point, remains incomplete, and that protecting the mental sphere requires a multilayered and context-sensitive strategy, informed by inter-American principles, PARLATINO’s model law, and UNESCO guidance, rather than the uncritical importation of a single foreign model.

## 2 Neurorights: definition and core elements

Ienca (2021) defines neurorights as ethical, legal, social, or natural principles of freedom or entitlement related to the cerebral and mental domain of the person; in other words, fundamental normative rules aimed at protecting and preserving the human brain and mind. This definition is significant because it shifts the focus of the debate. It is not merely about protecting a new type of data, but about safeguarding the mental sphere as an autonomous dimension of personality. In the most influential framework today, this field is organized around four fundamental axes (Ienca, 2021):

- Cognitive liberty, understood as the power of self-determination over one’s own mental processes, including the use or refusal of neurotechnologies;
- Mental privacy, that is, protection against access, collection, inference, circulation, and undue exploitation of information concerning mental states and brain activity;
- Mental integrity, aimed at preventing external interventions capable of harming, altering, or improperly exploiting the subject’s neural functioning;
- Psychological continuity, directed at preserving personal identity and the narrative coherence of psychic life in the face of profound technological interferences.

This taxonomy is analytically useful, but it should not be treated as conceptually closed or dogmatically mandatory. Part of the literature has therefore suggested that neurorights are better understood as reconceptualized human rights, that is, as a set of protections that derive from, update, and specify pre-existing guarantees in light of neurotechnological risks. Under this view, the legal task is to preserve what is normatively distinctive about the mental sphere without inflating the catalogue of rights or assuming, without sufficient justification, that every neural risk is legally exceptional (Ienca and Andorno, 2017; Bertoni and Ienca, 2024; Muñoz and Marinaro, 2023; Borbón et al., 2020; Tasioulas, 2021; Gaitán Uribe, 2017; Brown, 2024).

These categories demonstrate that the legal problem is not exhausted by the protection of data as an informational attribute. The core of the issue lies in the relationship between the brain, deliberation, and power. For this reason, the classical framework of personality rights and data protection, although indispensable, must be further developed to address the continuous capture of neural data, inferences about mental contents, and techniques of cognitive modulation (Ienca, 2021; López-Silva and Madrid, 2021).

In summary, neurorights represent an attempt to respond to a material transformation in the object of legal protection. If technology is already capable of turning the mind into a source of data and, in some cases, into a direct target of intervention, then the protection of human dignity must more explicitly encompass psychic autonomy, mental privacy, and the integrity of the decision-making process.

## 3 The Chilean case

Chile was the first country to introduce, at the constitutional level, a specific clause for the protection of brain activity and the information derived from it, through Law No. 21.383/2021, which amended Article 19(1) of the Chilean Constitution. The reform established that scientific and technological development must serve individuals and respect their lives and physical and mental integrity, and that the law shall provide special protection for brain activity and the information derived therefrom (Chile, 2021).

The literature describes this amendment as an inaugural milestone in the constitutionalization of neurorights, while also highlighting conceptual ambiguities and disputes regarding the scope of the reform. On the one hand, authors such as Ruiz et al. (2024) emphasize the paradigmatic value of the Chilean experience, which shifts the protection of the mind to the realm of fundamental rights and places the issue within contemporary constitutional debate. On the other hand, López-Silva and Madrid (2021) question whether it is truly necessary to create autonomous rights, arguing that part of this protection could be subsumed under existing frameworks of privacy, mental integrity, and personal data protection.

This dichotomy between normative innovation and the sufficiency of traditional categories gained concrete expression in the case Girardi v. Emotiv. The controversy involved the Insight device, an electroencephalography headset marketed by the company Emotiv. In examining the case, the Chilean Supreme Court emphasized the need for specific and informed consent for the processing of brain data, as well as the risks of reidentification, unauthorized reuse, surveillance, and the commercialization of neurodata. The case is emblematic because it demonstrates that the traditional data protection framework, on its own, does not satisfactorily address the challenges posed by brain information, precisely due to its inferential nature and its close connection to mental privacy (Cornejo-Plaza et al., 2024).

At the same time, the Chilean experience is not immune to criticism. The literature has questioned whether the constitutional amendment adds genuinely new protection or whether it reproduces protections already implicit in privacy, mental integrity, physical integrity, freedom of thought, and informational self-determination. Other criticisms concern the risk of conceptual vagueness, neuroessentialist assumptions, rights inflation, and the possibility that highly open-ended provisions may generate redundancy, interpretive uncertainty, or excessive restrictions on research if translated into overbroad statutory rules. In this sense, Chile is important not because it settles the debate, but because it makes visible the tension between symbolic constitutional innovation and the need for precise, operational, and scientifically calibrated regulation (Zúñiga Fajuri et al., 2020; López-Silva and Madrid, 2021; Borbón et al., 2020; Bublitz, 2022; Hertz, 2023; Ruiz et al., 2024).

In comparative terms, Chile operates as a normative laboratory. Its main contribution lies not only in having “created” new rights, but in recognizing that brain activity and the information derived from it possess a distinct sensitivity, thus requiring enhanced protection. This move is globally significant because it shifts the debate from the generic rhetoric of privacy to a more specific discussion on the protection of the human mind in the digital age.

## 4 Latin American regulatory pathways beyond Chile

Broadening the comparison beyond Chile is important because Latin America is already experimenting with different regulatory architectures. Mexico, for example, has pursued a more articulated pathway combining a proposed constitutional reform with a Draft General Law on Neurorights and Neurotechnologies. Rather than relying exclusively on constitutional symbolism, this model seeks to connect neuroprotection with a broader federal legislative structure, including health regulation, cybersecurity, and criminal law, thereby building a transversal framework oriented toward cognitive dignity and the governance of neurotechnological risks. Although still embryonic, the Mexican debate shows that the regional discussion is not exhausted by the Chilean model and that Brazil may learn from alternative legislative designs within the global south (Mexico, 2024; Vázquez Azuara and Zendejas Conde, 2024).

A different pathway can be observed in Argentina. At the federal level, legislative proposals have focused on procedural safeguards, especially judicial authorization and informed consent for the forensic use of neurotechnologies, while the Province of Misiones has adopted a concise but innovative subnational law protecting neural activity, neurodata, mental autonomy, and neurocognitive integrity. These initiatives are relevant because they illustrate two additional lessons for Brazil: first, that neuroregulation may develop through sector-specific and procedural mechanisms rather than only through constitutional reform; and second, that rules on evidence, consent, confidentiality, therapeutic limits, and protection of neural data may be as important as the formal recognition of new rights (Argentina, 2022; Province of Misiones, 2023; Vázquez Azuara and Zendejas Conde, 2024).

## 5 The Brazilian scenario under construction

In Brazil, there is currently no specific legislation on neurorights. The issue has been addressed in a fragmented manner through constitutional, civil, digital, and data protection norms, as well as legislative initiatives that signal a growing recognition of the problem. According to a consultation conducted on April 7, 2026, on the websites of the Chamber of Deputies and the Federal Senate, Bill No. 2,174/2023, Bill No. 522/2022, and Constitutional Amendment Proposal No. 29/2023 were still under consideration.

Bill No. 2,174/2023 is normatively ambitious because it gathers, in ordinary legislation, an extensive catalogue of guarantees and prohibited conducts related to the brain and the nervous system. However, it should not be described as systematic without qualification. Its architecture hybridizes rights, principles, programmatic commitments, and regulatory prohibitions, and still requires clearer dogmatic distinctions between direct neural intervention, neural-data processing, mental-state inference, manipulation of decision-making, and violations of personality rights. This matters because the legal goods at stake may require different remedies in constitutional, civil, administrative, procedural, and criminal law. Even so, the bill’s practical importance is undeniable, since it identifies concrete prohibited conducts—such as forced brain modification, non-consensual mind reading, unauthorized brain monitoring, and non-consensual brain manipulation—that could serve as a starting point for a more precise architecture of protection.

Bill No. 522/2022 follows a different regulatory path. Rather than creating an autonomous neurorights microsystem, it seeks to insert neural data into the logic of Brazilian data-protection law. The contrast between Bills No. 2,174/2023 and 522/2022 is analytically important because it reveals the coexistence of at least two strategies in Brazil: a broader neurorights statute and an incremental adaptation of pre-existing data-protection law. Constitutional Amendment Proposal No. 29/2023 keeps the constitutional debate open, but the Brazilian scenario remains marked by fragmentation and by the absence of a consolidated operative regime.

A further limitation of the Brazilian debate is that it still lacks a sufficiently articulated procedural and criminal-law dimension. If neural evidence can be used to infer mental states, memory patterns, intentions, or vulnerabilities, future regulation will need to distinguish administrative data protection from forensic admissibility, criminal prohibition, evidentiary exclusion, judicial authorization, and safeguards against coercive or hidden extraction of neural information (Brasil, 2022a,b, 2023a,b; PARLATINO, 2023; Mexico, 2024).

The opinion issued by the Health Committee, however, shifts the discussion in another direction. Instead of consolidating a distinct microsystem for neural data, the substitute bill seeks to accommodate the issue within the existing logic of the LGPD (Lei Geral de Proteção de Dados) (Brasil, 2018), aligning it with the regulatory framework applicable to sensitive data and health data. This shift is symptomatic, as it reveals the oscillation between two regulatory strategies: creating an autonomous regime or adapting the existing data protection framework (Brasil, 2024).

In turn, Constitutional Amendment Proposal No. 29/2023 seeks to introduce a clause into Article 5 of the Federal Constitution to protect mental integrity and ensure algorithmic transparency. Its symbolic merit is considerable: the proposal translates, into constitutional language, the perception that the protection of personal data, although necessary, does not exhaust the problems arising from the convergence between artificial intelligence and neurotechnology. At the same time, the very existence of the proposal indicates that the current constitutional text does not yet provide a sufficiently explicit response for the protection of autonomy.

Accordingly, the Brazilian scenario reveals discursive advancement, but not normative consolidation. Compared with Chile, Brazil adopts a gradual, fragmented, and multi-source approach, distributed across ordinary legislative bills, attempts at constitutional reform, and possible reinterpretation of the LGPD (Brasil, 2018). This plurality may foster regulatory flexibility, but it also prolongs uncertainty regarding the legal status of neural data and the permissible limits of technological intervention on the human mind.

## 6 Discussion

It is argued here that the current Brazilian legal framework is insufficient to guarantee neurorights. This does not mean that there is no applicable protection. On the contrary, the Constitution, the Civil Code (Brasil, 2002), the Brazilian Civil Rights Framework for the Internet (Marco Civil da Internet) (Brasil, 2014), and the LGPD (Brasil, 2018) form a relevant protective mosaic. The problem lies elsewhere: their concepts, triggering conditions, and institutional remedies were designed to address privacy, personality rights, communications, and personal data in the classical sense, rather than the capture, inference, and modulation of mental life by neurotechnological systems.

The Brazilian Constitution protects intimacy, private life, and the confidentiality of data and, since Constitutional Amendment No. 115/2022, also ensures the fundamental right to the protection of personal data, including in digital environments. These guarantees are essential, but they remain too generic given the neurotechnological risks. The reason is straightforward: the mental sphere is not reducible to communication, ordinary data, or a mere extension of classical privacy. Neurotechnologies can capture brain signals, extract probabilistic correlations, infer emotional states, or direct stimuli in ways that alter preferences without necessarily violating, in any evident manner, the confidentiality of communications or private life as traditionally conceived (Brasil, 1988; Constitutional Amendment No. 115/2022; Ienca, 2021).

Moreover, the Brazilian constitutional text does not expressly formulate guarantees equivalent to cognitive freedom, mental privacy, mental integrity, and psychological continuity. This does not imply a complete absence of protection; rather, it indicates protection by means of interpretative derivation. In such a sensitive domain, relying exclusively on hermeneutical inferences increases legal uncertainty, hinders preventive administrative action, and shifts to the Judiciary the task of constructing, on a case-by-case basis, limits that could instead be established in advance by the legislature.

The LGPD (Brasil, 2018) is a central component of the Brazilian digital legal framework, but its usefulness for neurorights is only partial. The first problem lies in its legal typology. Article 5, II, of the LGPD (Brasil, 2018) enumerates sensitive personal data and does not expressly mention neural data. Although it is possible, in certain cases, to classify such data as health data, biometric data, or as a sensitive category through expansive interpretation, this solution is contingent and insufficiently precise for a type of technology capable of accessing memory, attention, emotion, intention, or cognitive traits. The very fact that Congress is debating Bill No. 522/2022 demonstrates that the current wording of the law does not satisfactorily resolve the issue.

Even when neural data are treated as sensitive, the regime established under Article 11 of the LGPD (Brasil, 2018) was not designed for the singularity of mental life. The law permits the processing of sensitive data without consent in situations such as legal obligation, public policies, research, the regular exercise of rights, health protection, and fraud prevention. These legal bases may be reasonable in various contexts, but they become excessively broad when the data at issue are capable of exposing mental content or enabling highly intrusive neurocognitive profiling. In the context of neural data, the risk is not only improper disclosure; it also lies in the inferential exploitation of subjectivity (Brasil, 2018; Cornejo-Plaza et al., 2024).

There is also a deeper structural limitation: the LGPD (Brasil, 2018) primarily governs data processing, not intervention upon the mind. It does not directly regulate techniques of neuromodulation, persuasive design coupled with neural signals, continuous monitoring of cognitive states in the workplace or in education, or the inferential manipulation of preferences by systems that learn from brain-based feedback. In other words, neurotechnological harm is not merely informational; it may also be identity-related, volitional, and integrative.

Finally, Article 20 of the LGPD (Brasil, 2018), by ensuring the right to review automated decisions, provides a form of protection that is both ex post and limited. The provision operates only after an automated decision has already affected the data subject’s interests. Much of the neurotechnological risk, however, arises prior to that moment: in the opaque collection of neural signals, in the production of inferences about personality, in behavioral personalization, and in the induction of choices. Within this interval, the LGPD does not provide sufficient normative language to protect cognitive freedom or psychological continuity.

Personality rights as provided in the Civil Code (Brasil, 2002) also offer important protection, particularly by affirming their inalienability and non-waivability, as well as the inviolability of private life. However, their predominant logic is reactive: cessation of harm, injunctive relief, and civil reparation after the occurrence or imminence of damage. This repertoire is relevant, but it does not amount to a regulatory regime capable of imposing prior technical standards of safety, enhanced consent, algorithmic auditability, the segregation of therapeutic and commercial uses, or specific governance for brain–computer interfaces (Brasil, 2002; Cornejo-Plaza, 2023).

The relationship between identity and personality also deserves separate clarification. If neurotechnological risks affect the continuity of the self, they do not concern only data protection; they also concern the legal expression of personhood. In the literature reviewed here, mental privacy is linked to narrative and relational identity because the self is constituted through processes of self-interpretation, communication, and social recognition. For this reason, interventions that read, profile, or alter mental states may simultaneously affect identity in a philosophical sense and personality in a legal sense, which helps explain why Brazilian personality rights are relevant but not, by themselves, sufficient (Ienca and Andorno, 2017; Fernández, 2012; Wajnerman Paz, 2021; Brasil, 2023a,b).

A further difficulty is that regulation should not be driven by speculative alarmism. The Chilean debate has shown how neuroessentialism, neuroexceptionalism, and neurohype can create an artificial sense of urgency and obscure important distinctions between neuroimaging, neuromodulation, brain–computer interfaces, and other forms of digital inference. A sound Brazilian framework must therefore remain attentive to plausible harms without converting science-fiction scenarios into immediate legislative premises. This neuroethical caution does not weaken protection; rather, it supports more proportionate rules, better scientific calibration, and a more credible relationship between innovation and precaution (Alegre, 2017; Ligthart et al., 2019; Ruiz et al., 2024; Gilbert, 2024).

At the same time, avoiding neurohype does not mean ignoring the need for governance. Inter-American and global instruments already offer useful benchmarks for a more mature Brazilian strategy. The Inter-American Juridical Committee’s declarations, PARLATINO’s model law, and UNESCO guidance point to minimum safeguards such as purpose limitation for neural and mental data, reinforced protection for sensitive information, informed consent, non-discrimination, special caution in criminal proceedings, judicial authorization for coercive access, and institutional oversight. In addition, the Chilean experience of public deliberation around Congreso Futuro illustrates the value of combining expert dialogue, legislative debate, and public visibility before adopting a final statutory model. Brazil would therefore benefit from a broader consultative process prior to regulatory consolidation (Inter-American Juridical Committee, 2021, 2023; PARLATINO, 2023; UNESCO, 2025).

A similar situation arises with the Brazilian Civil Rights Framework for the Internet (Marco Civil da Internet). Articles 7 and 10 protect intimacy, private life, communications, and personal data within the internet ecosystem, but the statute was designed to regulate relationships mediated by providers and applications. Neurotechnologies, however, also manifest as wearable devices, sensors, embedded software, medical devices, productivity tools, and hybrid systems that do not fit seamlessly within the sectoral architecture of the Marco Civil. Moreover, the statute does not address issues such as the decoding of mental states, cognitive manipulation, the cybersecurity of neurodevices, and the labor or educational uses of neural data (Brasil, 2014).

For these reasons, protecting the mental sphere in Brazil should not be reduced to a binary choice between creating entirely new rights and relying exclusively on pre-existing guarantees. A more plausible route is a multilayered framework in which the Constitution, the LGPD, the Civil Code (Brasil, 2002), and the Marco Civil da Internet continue to function as a baseline, while legislation and regulation progressively specify concepts such as neural data, mental data, cognitive biometrics, brain–computer interfaces, enhanced consent, impact assessment, and sector-specific limits on therapeutic, commercial, educational, labor, security, and criminal-procedural uses. Within such a framework, the law would also need to delimit the legal interests that deserve heightened protection—especially brain activity, mental privacy, mental identity, psychological continuity, and decisional self-determination—and to clarify, where appropriate, the civil, administrative, procedural, and criminal consequences of non-consensual reading, monitoring, hacking, inference, or manipulation of mental activity (Ienca and Malgieri, 2022; Muñoz and Marinaro, 2023; Magee et al., 2024; Brasil, 2022a,b, 2023a,b; Mexico, 2024).

Such a framework would also preserve the most valuable lesson of the Chilean case without mechanically reproducing its constitutional design. Brain activity and information derived from it may justify heightened protection, but that protection should be operational, proportionate, and institutionally coordinated. In the Brazilian context, this means combining constitutional interpretation, data protection, personality rights, administrative oversight, public consultation, and, where necessary, specific legislation capable of prohibiting non-consensual reading, monitoring, inference, hacking, and manipulation of mental activity with sufficient conceptual precision. Chile remains an important starting point, but the Brazilian debate should be shaped by a broader Latin American repertoire and by Brazil’s own constitutional, dogmatic, and institutional culture (López-Silva and Madrid, 2021; Ruiz et al., 2024; Brasil, 2023a,b).

## 7 Conclusion

The emergence of neurorights reflects a material transformation in the object of legal protection: what is at stake is no longer merely the body or data, but technological access to mental life itself. Accordingly, the central question is whether the current legal framework is capable of ensuring protection against the specific risks posed by neurotechnology. The answer advanced in this article is negative.

The Chilean experience demonstrates that the protection of brain activity can be elevated to the constitutional level and produce significant interpretative effects, including concrete application, as illustrated by the case Girardi v. Emotiv. Brazil, in turn, already acknowledges the issue through initiatives such as Bill No. 2,174/2023, Bill No. 522/2022, and Constitutional Amendment Proposal No. 29/2023, but has not yet translated this recognition into specific positive law.

The comparative picture also suggests that Latin America is developing several, not one, regulatory grammars. Chile emphasizes constitutional recognition; Mexico has moved toward a combined constitutional-and-general-law model; Argentina and Misiones illustrate procedural and subnational experimentation; and Brazil remains in a stage of dispersed legislative construction. The lesson is therefore not that Brazil should copy Chile, but that it should identify which combination of constitutional, statutory, procedural, administrative, and deliberative tools best fits its own legal culture and institutional capacities (Mexico, 2024; Vázquez Azuara and Zendejas Conde, 2024; Argentina, 2022; Province of Misiones, 2023; Brasil, 2023a,b; Inter-American Juridical Committee, 2023; PARLATINO, 2023).

A Brazilian response to neurotechnological risks should thus move beyond the false choice between merely importing a foreign neurorights model and doing nothing until harms become widespread. The more defensible position is to adopt a multilayered framework for the protection of the mental sphere: one that strengthens constitutional interpretation, refines data protection, clarifies the role of personality rights, develops proportionate sector-specific safeguards, promotes public consultation inspired by experiences such as Chile’s Congreso Futuro, and, where clearly justified, creates specific legal prohibitions and remedies for non-consensual interference with brain and mental activity. In this sense, Chile remains an important starting point, but not an exclusive template for Brazilian regulation. What is needed is a Brazilian framework that is comparatively informed, conceptually restrained, attentive to neuroethical caution, and institutionally adapted to the country’s constitutional and regulatory architecture (Muñoz and Marinaro, 2023; Magee et al., 2024; López-Silva and Madrid, 2021; Brasil, 2023a,b).

## Data Availability

The original contributions presented in the study are included in the article, further inquiries can be directed to the corresponding author.
